# Resveratrol oligomer structure in Dipterocarpaceaeous plants

**DOI:** 10.1007/s11418-020-01412-x

**Published:** 2020-04-30

**Authors:** Tetsuro Ito

**Affiliations:** 1 Laboratory of Pharmacognosy, Gifu Pharmaceutical University, 1-25-4 Daigaku-nishi, Gifu, 501-1196 Japan; 2 Present Address: Laboratory of Pharmacognosy, Department of Pharmacy, Faculty of Pharmacy, Gifu University of Medical Science, 4-3-3 Nijigaoka, Kani, Gifu, 509-0293 Japan

**Keywords:** Dipterocarpaceae, Resveratrol, Oligomerization, Structural diversity

## Abstract

Oligostilbenoids are a group of natural products derived from the oxidative coupling of C_6_–C_2_–C_6_ units found in some plant families. A structurally diverse chemical pool is produced after the successive regioselective and stereoselective oligomerization of resveratrol. This review describes the current status and knowledge of the structure of resveratrol oligomers (ROs) in Dipterocarpaceaeous plants (DPs). Beginning with the recently validated formation of ROs in DPs, each downstream conversion is described from the perspective of the resveratrol coupling mode. Particular emphasis is placed upon the regioselectivity of monomer- and dimer-derived radical–radical coupling processes, which are responsible for producing dimers, trimers, and tetramers with various cyclic frame skeletons, as well as related processes that result in highly condensed scaffolds, such as hexamers and octamers. Trimers in oxidized, dearomatized, and rearranged forms are also summarized, as well as the biogenic relationship between the compounds. Furthermore, emphasis is placed on the *O*- and *C*-glucosides of ROs, as well as on the hetero-coupled ROs. In addition, several stereoisomers that originate from asymmetric carbons and the stereochemistry with respect to the conformation due to the chiral axis are described. Besides, NMR spectroscopic properties such as coalescence and anisotropy are briefly described. Approaches to determine absolute configuration are also summarized.

## Introduction

**Fig. 1:**
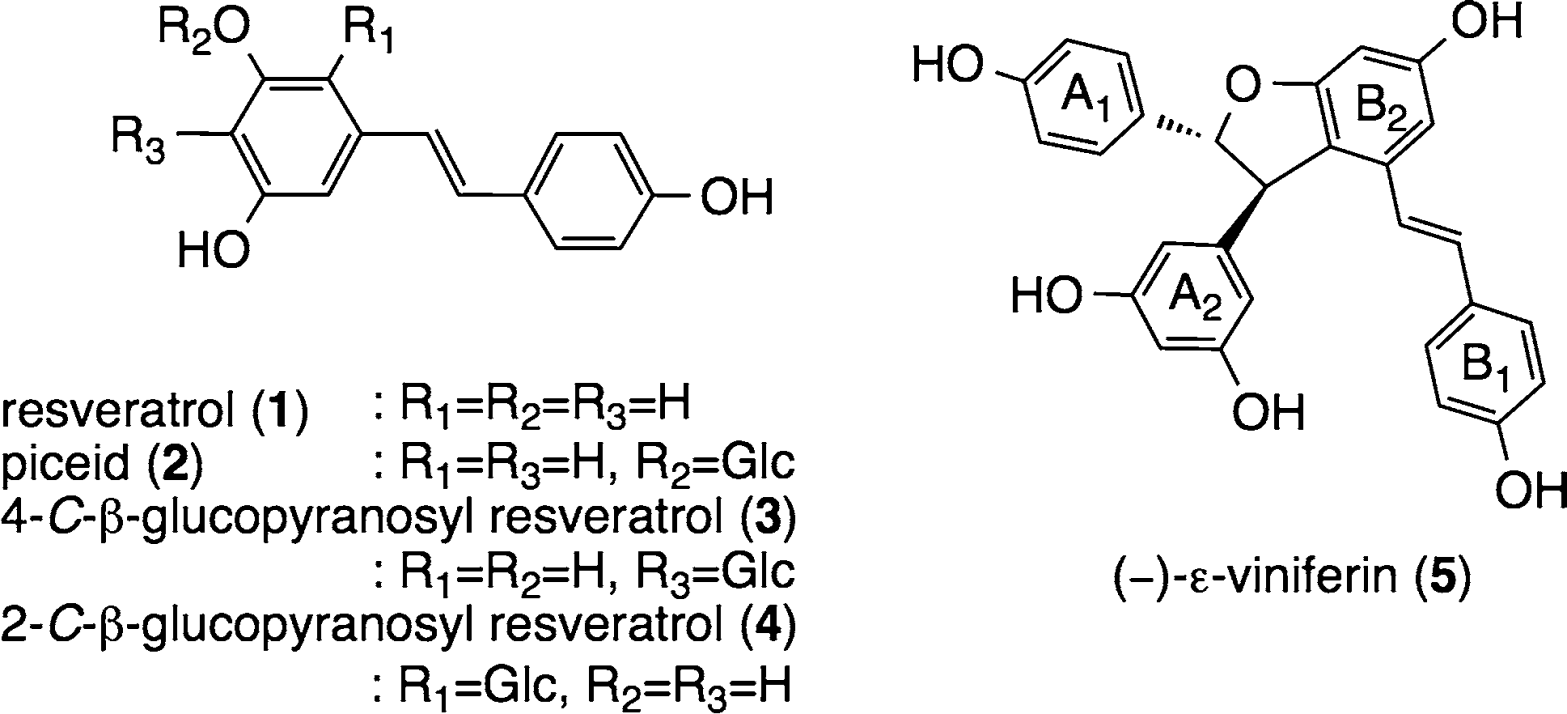
Structures of monomeric resveratrol derivatives (**1**–**4**) and **5**

In the plant kingdom, resveratrol oligomers (ROs) can be found in a number of plant families, such as the Dipterocarpaceae, Vitaceae, Cyperaceae, Fabaceae, Paeoniaceae, and Gnetaceae families [1–4]. The Dipterocarpaceaeous plant (DP) is the dominant plant family of Southeast Asia, with a total of 470 species [5, 6]. Indeed, plants in this family are a rich source of ROs, which are produced from the successive condensation of resveratrol (**1**: *trans*-3,5,4′-trihydroxystilbene) (Fig. 1). The first RO was characterized from *Hopea odorata* in 1966 [7]; in the following 25 years, dozens of structurally related compounds have been identified [1]. In recent years, several hundred ROs have been isolated from DPs with their structures determined accordingly [2]. In essence, this structural diversity stems from patterns of phenoxy radical–radical coupling that yield various fused-ring systems containing asymmetric carbons, which, in turn, give rise to regioisomerism and stereoisomerism. Structural diversity is further expanded by divergent structural modifications, such as oxidation, dearomatization, substituent rearrangement, and glucosylation [8]. Since wide-ranging bioactivity screens have been applied to RO and, in the process, their various activities identified, it remains essential to expand the current chemical library as well as to identify the exact structure of each isolate.

Scientific interest in ROs in DPs began in 1999. The impetus for this interest was due to a number of reasons. First, discovering the remarkable biological properties of the building block (BB), resveratrol, in human-health protection [9] and plant defense [10, 11] was a significant discovery. Second, several reports suggested (but did not prove) that certain types of ROs are pathway intermediates to further condensed components [12–15]. Third, according to some reports, many ROs of widely differing skeletal types are optically active, suggesting unique and/or distinct coupling mechanisms. Finally, the challenging structural-elucidation work simply triggered academic interest. Initial research goals mainly encompassed two topics: to define the structural diversity afforded by the condensation degree, varying skeletons, and isomerisms due to the number of asymmetric carbons found in this class of natural products; to gain insight into the research area of polyphenol (i.e., the chemistry of RO), which requires expanding the existing chemical library and accumulating spectroscopic details. In the last 2 decades, phytochemical studies have achieved the isolation and structural elucidation of roughly 200 ROs in species belonging to the following seven genus: *Vatica* (*V. rassak* [16–19], *V. pauciflora* [20, 21], *V. albiramis* [22–25], and *V. chinensis* [25–27]), *V. bantamensis* [25], *Vateria* (*V. indica*) [28–33], *Upuna* (*U. borneensis*) [34–41], *Cotylelobium*, (*C. lanceolatum*) [42–44], *Dipterocarpus* (*D. grandiflorus*) [45, 46], *Shorea* (*S. hemsleyana* [33, 47–50], *S. uliginosa* [33, 51–54], and *S. cordifolia* [55]), and *Hopea* (*H. parviflora* [56–58] and *H. utilis* [59–61]). This review examines the structural aspects of RO, focusing on the derivatives mainly determined in our laboratory, and outlines their biogenetic relationship in combination with presenting skeletal diversity. For supporting information, a comprehensive list of RO in DP and diverse biological properties are summarized by Rivière et al. [4] and Keylor et al. [8], respectively.

## Definition

Resveratrol can be widely found in the plant kingdom and, in particular, in the products of the phenylpropanoid pathway; it is responsible for transforming phenylalanine into 4-coumaroyl-CoA, which finally enters the stilbenoid-biosynthesis pathway [62]. ROs are metabolites found in a small set of phylogenetically distant plant families [4], the BB of which (C_6_–C_2_–C_6_) is successively oligomerized after generating phenoxy radicals and highly active quinomethides (QM), followed by spontaneous regioselective radical–radical coupling, regiodivergent Friedel–Crafts reactions, nucleophilic trappings, and tautomerizations [8, 14].

ROs differ from most other polyphenols (e.g., flavonoids, pyrones, quinones, and their downstream products) by having comparatively less structural diversity due to small variations and the limited patterns of functional groups; by expanding the chemical pool by oligomerization, the production of various frame skeletons is ensured as well as the participation of *O*- or *C*-glucoside (monoglucoside of resveratrol: **2**–**4** (Fig. 1)) as a BB. Additionally, they differ among themselves with respect to the inversion of the configuration of asymmetric carbons, which originate from the C_2_ units of the BBs. This results in the isolation of hundreds of derivatives that have characteristic stereoisomeric structural motifs, such as dihydrobenzofuran-, indane-, and bicyclo-ring systems that are conserved across DPs [2].

The researcher-friendly designating scheme has been applied to standardize the two oxygenated aromatic rings of phenol (A_1_) and the resorcinol rings (A_2_) in each resveratrol unit, in combination with a numbering order that denotes 14 carbons (1a–14a) starting from A_1_. The next letter in the alphabet and the next numbering order are B_1_, B_2_, and 1b–14b, respectively, with regard to additional resveratrol units. When we explain the condensation modes of resveratrol units, such as regioselective radical–radical coupling, the numbering orders, 1–14 and 1′–14′, are applied (e.g., coupling modes 8–8′, 8–10′, and 3–8′) according to the molecular species in question.

## RO structure in DPs

**Fig. 2:**
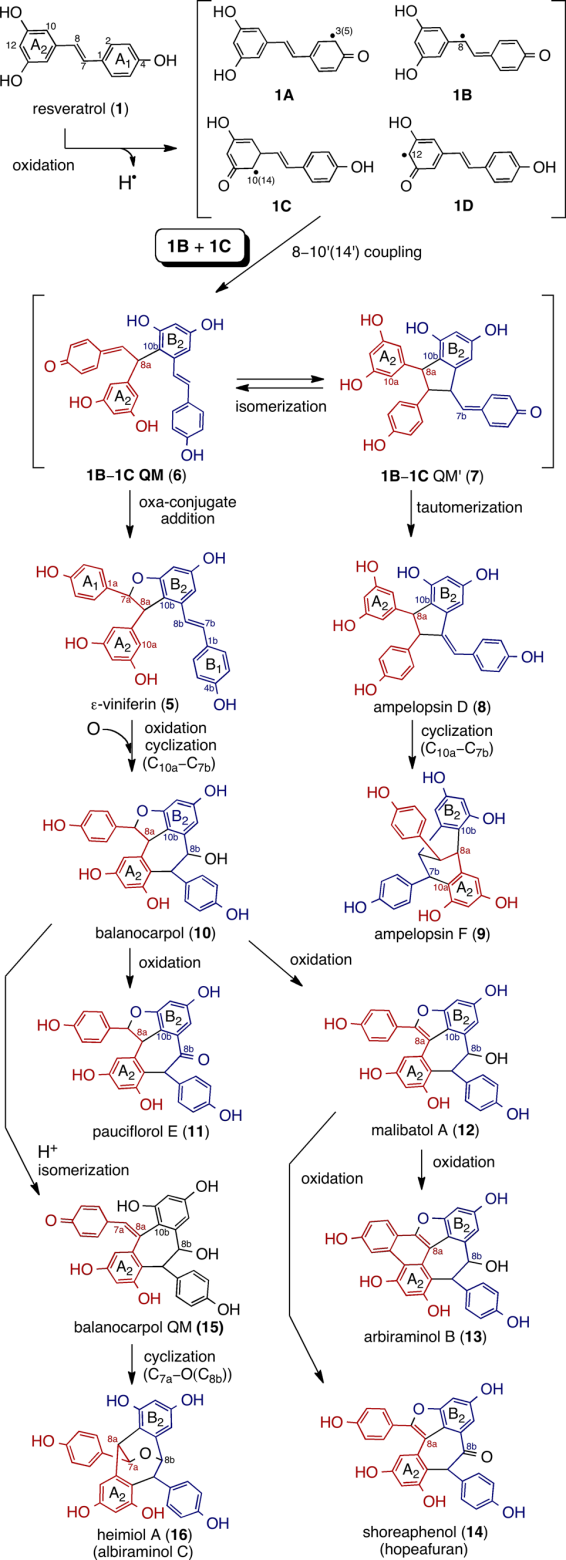
Plausible biosynthetic pathway of the 8–10′ dimers

**Fig. 3:**
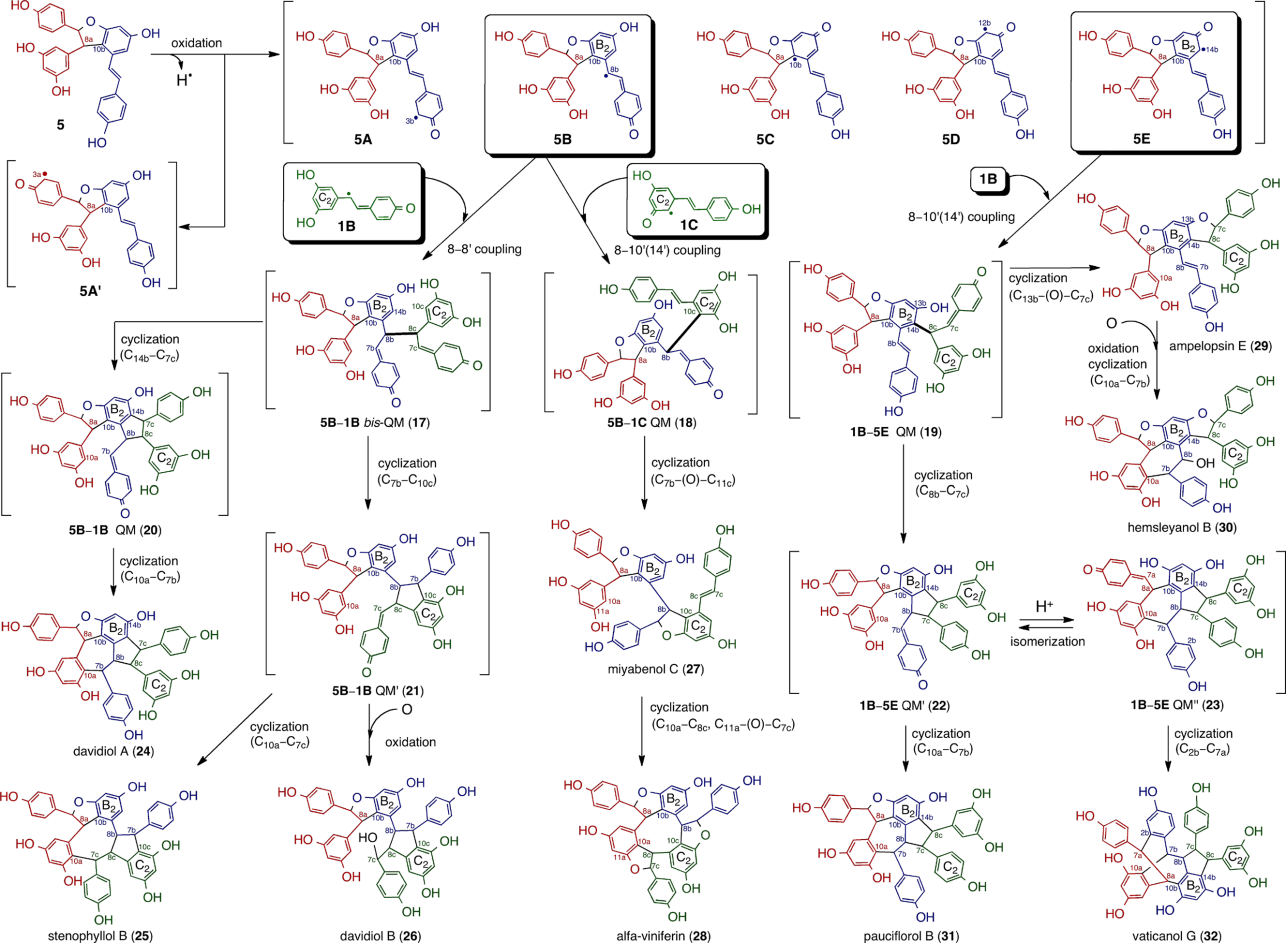
Plausible biosynthetic pathway of trimers produced via 8–8′ or 8–10′(14′) crossed coupling of **1** and **5**

**Fig. 4:**
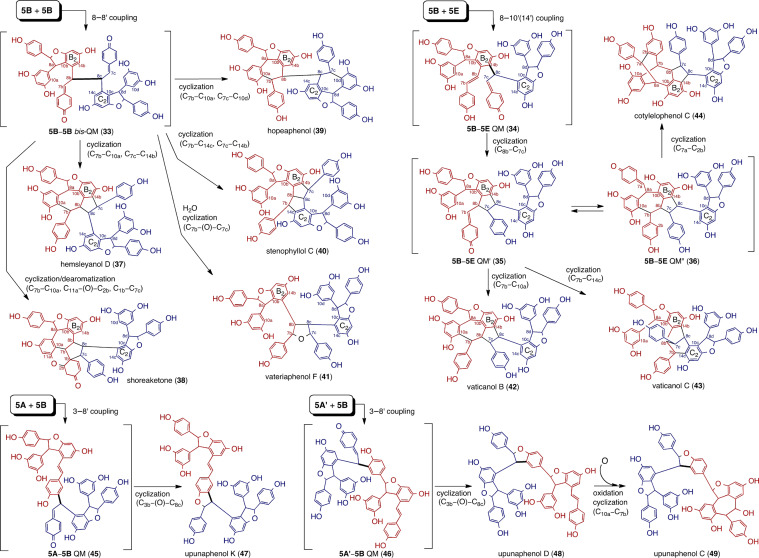
Plausible biosynthetic pathway of tetramers produced via the 8–8′, 8–10′(14′) and 3–8′ crossed coupling of the two molecules of **5**

Structurally diverse ROs can usually be found in planta as dimeric–tetrameric entities; to be sure, higher oligomers also exist. Because dimerization is the initial step in the global biosynthetic scheme of the chemical pool, it is crucial to clarify simple frame skeletons of the smallest oligomer to understand the further oligomerized (and more complex) skeletons of trimer–octamers lying downstream of biogenesis. ROs in DPs are regioselectively biosynthesized due to the coupling of oxidatively generated phenoxyl radicals (**1A**–**1D**), where the initial dimerization typically occurs through the 8–10′ coupling mode to produce various dimers that can be represented as (−)-ε-viniferin (**5**) (Figs. 1, 2) [11, 63]. The majority of resveratrol dimers are 8–10′ linked, but many different coupling modes exist in nature (e.g., 8–8′, 3–8′, and 8–12′); these coupling modes (and the potential substrates involved) often vary according to the species being analyzed [2]. The diverse reactivity of further generated reactive QM species, such as **5A**–**5E**, in combination with **1A**–**1D** further contribute to downstream regiodivergent reactions, which, in turn, results in the production of further condensed RO (Figs. 3, 4). In addition, the glucoside of **1** not only stores resveratrol in cell tissues and prevents it from being oxidized, but it also contributes to the biosynthesis of RO glucosides, which further expands their chemical diversity. This can be seen in the rich isolation of **2**–**4** (Fig. 1).

Generally, asymmetric carbons exist in proportion to the oligomerization degree, e.g., in many cases, the dimers and tetramers of resveratrol have four and eight chiral atoms, respectively. Among the 200 ROs isolated from the DPs, dimers, trimers, and tetramers are common. This trend can be observed in other families, including Vitaceae [1–4]; however, the further condensed derivatives, such as hexamers and octamers, are not common. Compound **5** is one of the most abundant dimeric resveratrols, which has been isolated from the majority of RO-containing plant families, such as Dipterocarpaceae, Vitaceae, Cyperaceae, Fabaceae, Paeoniaceae, and Gnetaceae, among which DPs are known to produce (−)-form. Indeed, this issue is vital in considering an absolute configuration of biogenetically downstream chemicals. Because the structural diversity of RO can be attributed to skeletal variations and the presence of stereoisomerism, analyzing two- and three-dimensional structures is an interesting challenge, academically speaking.

### Two-dimensional structures and the biosynthetic scheme of simply oligomerized resveratrol

**Fig. 5:**
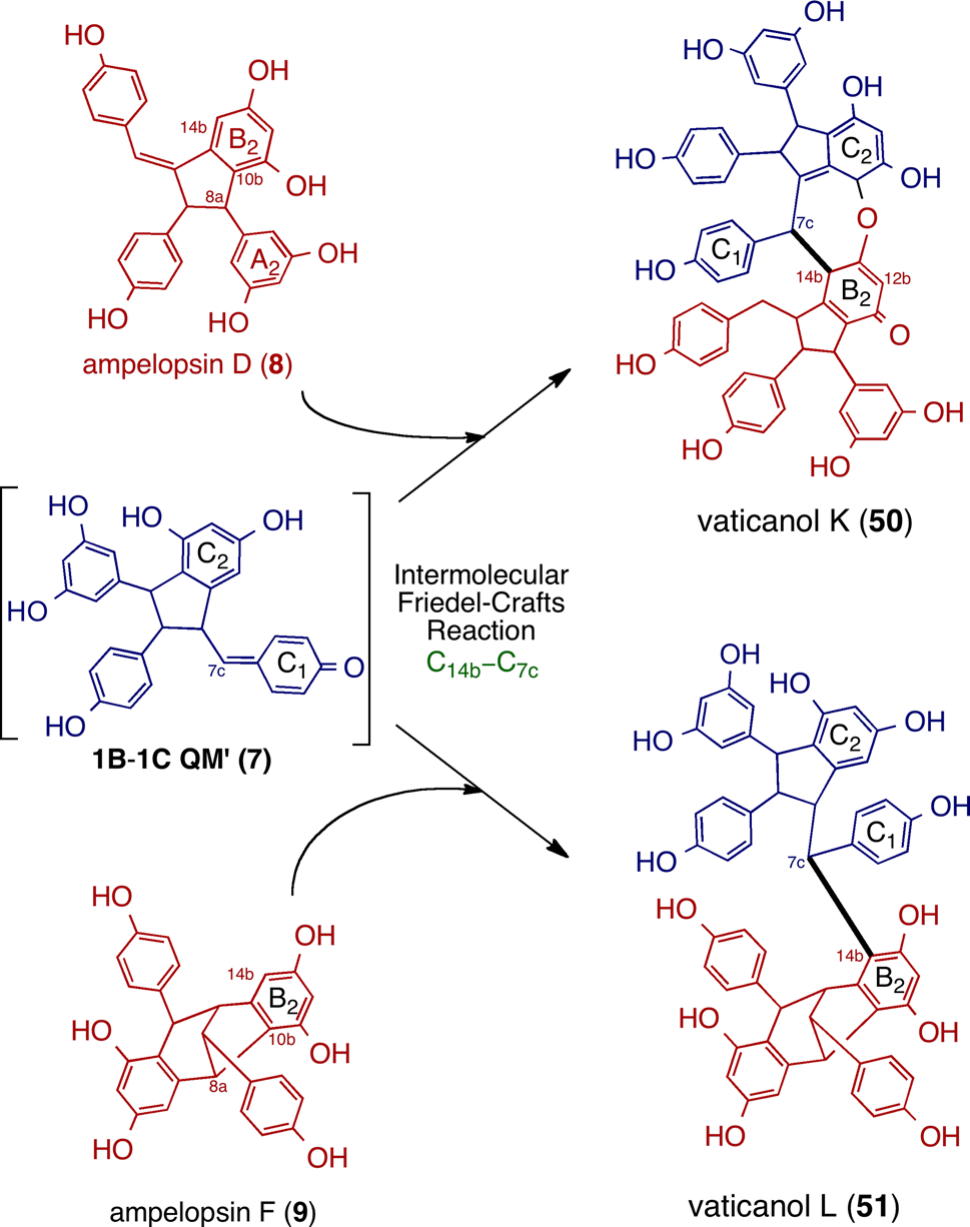
Tetramer formation via intermolecular trapping of quinone-methide intermediates

**Fig. 6:**
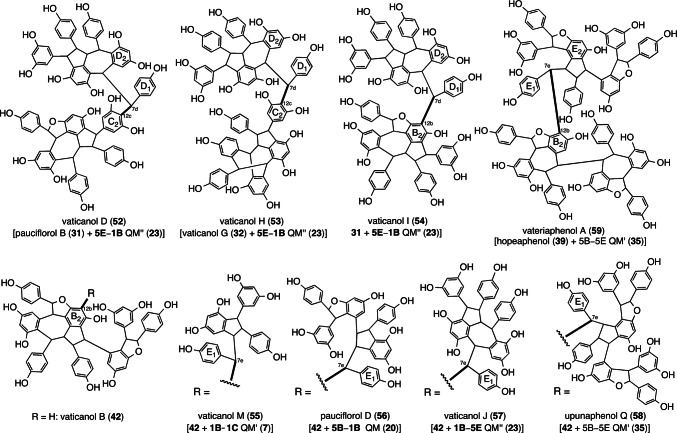
Structures of highly condensed RO

**Fig. 7:**
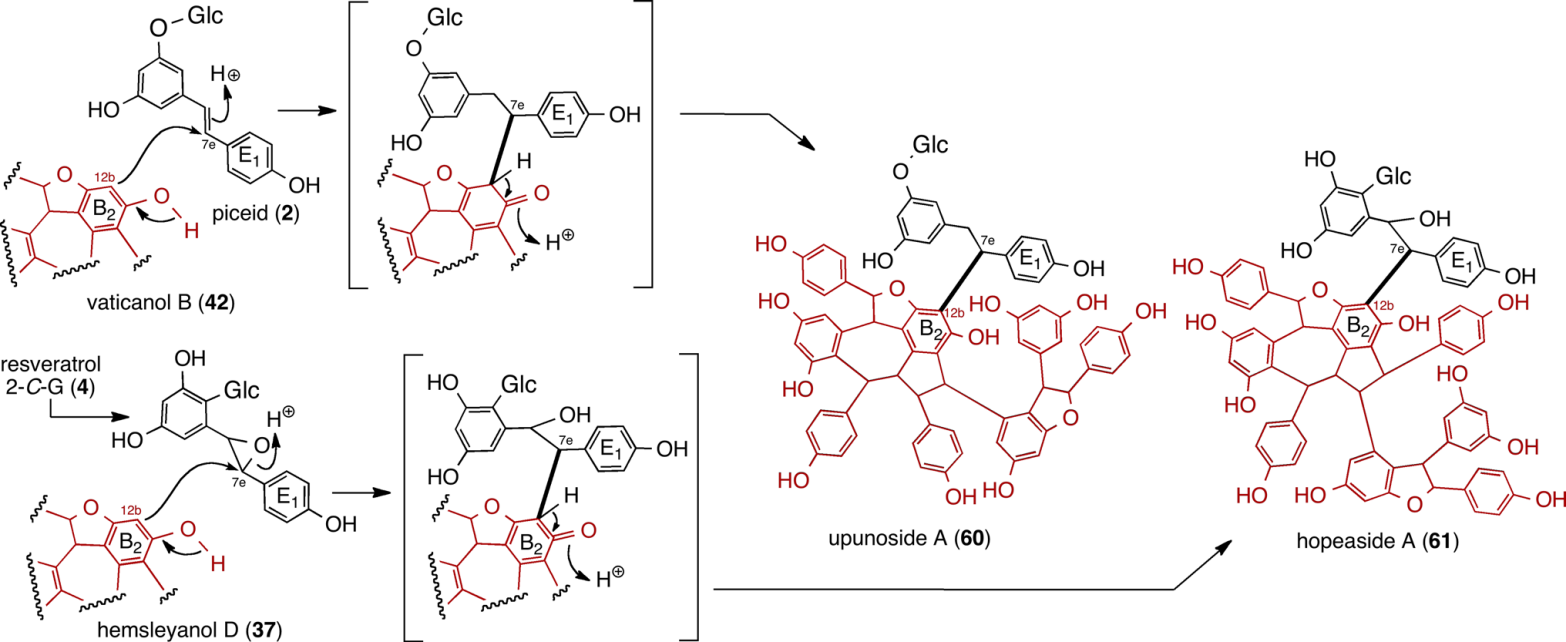
Proposed biosynthesis of *O*- and *C*-glucosides of pentamer

**Fig. 8:**
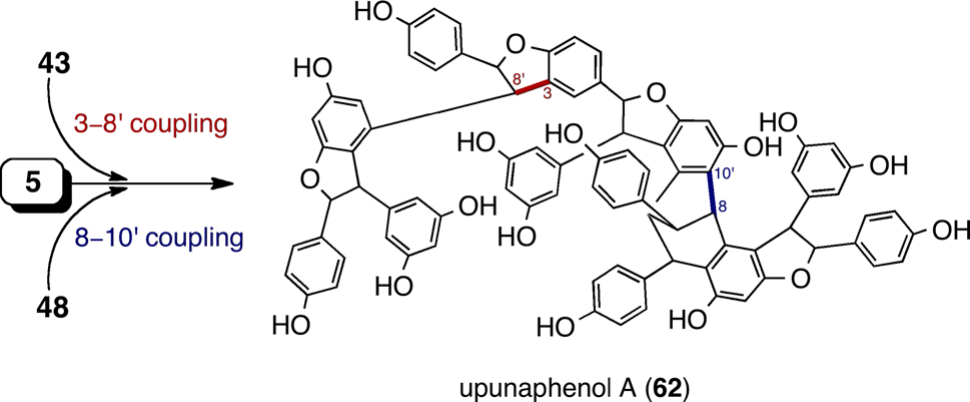
Proposed biogenesis of **62** through 3–8′ and 8–10′ coupling

In this section, representative ROs are discussed from the viewpoint of oligomerization degree and skeletal diversity with biosynthetic aspects. The listed compounds are prioritized to systematically depict a plausible biogenic relationship between compounds with topological differences. Indeed, our findings, which are based on experiments in the last 2 decades in combination with the existing literature, support the proposed biosynthetic aspects. In this section, each RO is delineated as a planar structure (Figs. 3, 4, 5, 6, 7, 8).

#### Resveratrol dimers

The resveratrol dimers in DPs bear 8–10′ C–C bonds, which stems from the initial coupling (Fig. 2). The key dimeric intermediate, **1B**–**1C**-QM (**6**), further undergoes particular cyclization(s) and tautomerization, thereby generating several different frame skeletons. As a result of the oxa-conjugate addition, dimers bearing heterocyclic dihydrobenzofuran (e.g., ε-viniferin (**5**)) are generated. Alternatively, 5-exo-trig cyclization through C_7a_–C_8b_ bond formation results in another QM (**1B**–**1C**-QM′ (**7**)), which undergoes further tautomerization and 6-exo-trig cyclization through the C_10a_–C_7b_ bond, which, in turn, results in resveratrol dimers with non-heterocyclic ring systems, such as indane (e.g., ampelopsin D (**8**) [64]) and bicyclo[3.2.1]octadiene (e.g., ampelopsin F (**9**) [65]).

Oxidation results in diverse oxidized 8–10′ dimers. This takes place due to the production of molecular species with dibenzocycloheptane skeletons (e.g., balanocarpol (**10**) [66]) upon the epoxidation of **5** and the intramolecular 7-exo-trig cyclization through the C_10a_–C_7b_ bond. Compound **10** acts as an intermediate to generate divergent oxidative products, such as pauciflorol E (**11**) [20], malibatol A (**12**) [67], arbiraminol B (**13**) [23], and shoreaphenol (**14**) [68] (hopeafuran [61]) (Fig. 2). A ketone (**11**) is produced when the direct benzylic-alcohol oxidation of **10** proceeds. Alternatively, an extended conjugated system is formed due to the formation of benzofuran through the two-electron oxidation of **10**, thereby yielding **12**. Further two-electron oxidation and the direct benzylic-alcohol oxidation of **12** results in **13** and **14**, respectively. Heimiol A (**16)** [69] (albiraminol C [22]) is a structural isomer of **10**, which is presumably derived through the intramolecular etherification of the C_7a_–O_8b_ bond due to the acid-mediated formation of para-quinone methide (**15)**.

#### Resveratrol trimers

Resveratrol trimers are presumed to be the products of the crossed coupling of **1** and **5**, which occur between two reactive QM species (**5B**/**5E** and **1B**/**1C**) via the 8–8′ or 8–10′(14′) mode. The initially generated QMs (**17**–**19**) proceed through further regiodivergent cyclization to form various molecular species, with the subsequently generated QMs (**20**–**23**) acting as the presumed biosynthetic intermediates for a diverse series of resveratrol trimers (**24**–**32**) (Fig. 3).

Trimers derived from **5B**–**1B** bis-QM (**17**) are referred to as “8–8′ trimers”, which is the naming scheme favored by Keylor et al. [8]. Although 8–8′ trimers are a minority in DP, QM (**17**) can still undergo cyclization via C–C bond formations, specifically C_14b_–C_7c_ and C_7b_–C_10c_ can be altered to form two QM intermediates: **5B**–**1B** QM (**20**) and **5B**–**1B** QM′ (**21**). This is supported by the existence of three ultimate products: davidiol A (**24**) [70, 71], stenophyllol B (**25**) [71], and davidiol B (**26**) [70]. Hitherto, a series of **5B**–**1B** coupled trimers has been isolated from *Vatica* and *Shorea* species [21, 50]; however, further phytochemical investigations must be conducted to examine whether this class of trimers is ubiquitous.

The reactive intermediate, **5B**, could also couple to the other resveratrol phenoxy radical, **1C,** via the 8–10′(14′) mode to produce **5B**–**1C** QM (**18**) as well as a series of 8–10′ trimers. This intermediate follows oxa-conjugate addition(s) to produce miyabenol C (**27**) [72] and α-viniferin (**28**) [73]. Several DPs belonging to the Shoreae family, such as *Shorea* and *Hopea,* produce **28** in rich quantities [50, 52, 74].

The production of plausible **1B**–**5E** QM (**19**) followed by cyclization and isomerization results in other QMs, specifically **1B**–**5E** QM′ (**22**) and **1B**–**5E** QM” (**23**). Indeed, the existence of said intermediates is strongly supported by the isolation of respective 8–10′(14′) trimers: ampelopsin E (**29**) [64], hemsleyanol B (**30**) [50], pauciflorol B (**31**) [21], and vaticanol G (**32**) [16]. In a fashion analogous to the biosynthesis of **10** via **5**, **29** can be altered to form **30**. Among these intermediates, **22** and **23** play a major role in the production of resveratrol trimers in several trimer-rich DPs classified as *Vatica* and *Cotylelobium* species [16, 19, 44]. Similar to the 8–10′ dimeric resveratrol, **5**, the downstream products of the 10–8′-coupled **1B–5E** are widely distributed among the various species of DP.

#### Resveratrol tetramers

The major production of resveratrol tetramers takes place after the dimerization of dimers, wherein **5** plays a central role, as shown in Fig. 4. In the majority of resveratrol tetramers, the structure can be attributed to the presence of the configurationally conserved dihydrobenzofuran motif, which is a crucial issue in explaining the absolute configuration of high-order ROs. The production of resveratrol tetramers proceeds in a similar fashion to the resveratrol trimers via the initial formation of two QMs, specifically **5B**–**5B** bis-QM (**33**) (8–8′ coupling mode) and **5B**–**5E** QM (**34**) (8–10′ coupling mode), which mechanistically correspond to **17** and **19** for resveratrol trimers, respectively. The QM intermediates, **33** and **34**, together with the further diversified QM scaffolds (**5B**–**5E** QM′ (**35**) and **5B**–**5E** QM" (**36**)) undergo numerous structural conversions, resulting in the production of a diverse chemical pool of 8–8′ and 8–10′ tetramers (**37**–**44**). Indeed, bicyclic structural motifs for **24**, **31**, and **32** can also be found in hemsleyanol D (**37**) [48], vaticanol B (**42**) [19], and cotylelophenol C (**44**) [42], respectively. This indicates the existence of a similar mechanism with corresponding QM intermediates between the resveratrol trimers and tetramers.

In the case of 8–8′-tetramer biosynthesis, the bis-QM (**33**) is altered through regiodivergent cyclization(s) and dearomatization, resulting in the production of **37**, shoreaketone (**38**) [33, 54], hopeaphenol (**39**) [7], and stenophyllol C (**40**)[71]. Molecules bearing the C_2_-axis of symmetry, formed via intramolecular cyclization (**39**: 2 × 7-exo trig; **40**: 2 × 5-exo trig), are the characteristics of the 8–8′ tetramers. The other C_2_ molecule, vateriaphenol F (**41**) [28], has an additionally introduced oxygen atom on the symmetrical plane, which forms a tetrahydrofuran ring. It can be assumed that the tetrahydrofuran ring forms via the double addition of water across **33**, followed by dehydrative cyclization. The biosynthetic mechanism for **38** involves subsequent cyclization via C–C bond formation (C_7b_–C_10_ and C_1b_–C_7c_) to give spirocyclic cyclopentane, which is followed by oxa-conjugate addition to produce a C–O bond (O_11a_–C_2b_). In turn, this results in the generation of the spirocyclohexene in the ultimate product [33]. Among 8–8′ tetramers, **39** [7] is one of the most abundant tetramers in DPs.

Alternatively, the biosynthesis of 8–10′ tetramers can be explained by another primary intermediate, **34**, which preferentially undergoes intramolecular 5-exo-trig cyclization through C_8b_–C_7c_ isomerizing to **35** instead of 7-exo-trig cyclization as in **39**, the latter of which would produce less types of frame skeletons than those from **33**. The QM (**35**) and isomerized **5B**–**5E** QM” (**36**) follow different cyclization pathways, resulting in the production of vaticanols B (**42**), C (**43**) [19] (vat

diospyroidol [75]), and **44** [42]. In fact, **42**, which is usually present with **43**, is another abundant tetramer found in many DPs. It is interesting that the 8–10′ tetramers produced via **35** are specifically isolated from DPs, which further undergo downstream modification resulting in the scaffold characteristics, such as high-order oligomers (e.g., hexamers and heptamers).

The 3–8′ connectivity is uncommon among the ε-viniferin-derived resveratrol tetramers. This is because there are fewer possible cyclization-reaction pathways compared with their 8–8′ and 8–10′ counterparts. Upon the oxidation of **5** and 3–8′ dimerization, hypothetical para-QM intermediates (**5A**–**5B** QM (**45**) and **5A′**–**5B** QM (**46**)) can follow oxa-conjugate addition to generate 3–8′ tetramers (upunaphenols K (**47**) [37] and D (**48**) [38]). The downstream analogues, such as upunaphenol C (**49**) [38] upon epoxidation and 7-exo-trig cyclization of **48**, are also minorities. Currently, an array of 3–8′ tetramers has only been found in the monotypic genus *Upuna borneensis*.

The other group of resveratrol tetramers is produced via 7–10′(14′) connectivity between two dimeric resveratrol units. The first isolation is vaticanol K (**50**) from *Vatica chinensis*, which possesses an unprecedented fused 2,7-dihydrooxepine-QM skeleton [27]. We presume that the plausible biogenesis, including the concerted intramolecular cyclization of two resveratrol dimers, is followed by desaturation extending conjugation. However, the further isolation of vaticanol L (**51**) with a 7–10′ bond suggests that the regioselective dimeric dimerization reactions occur due to the nucleophilic trapping of QM [26]. A similar scaffold of resveratrol tetramers with 7–10′(14′) bonds consists of cajyphenol A, which has been previously isolated from *Cayratia japonica* (Vitaceae) [76]. It has been proposed that cajyphenol A consists of metabolites after the cross coupling of quadrangularin A (8–8′ resveratrol dimer) [77] and its penultimate biosynthetic intermediate, which involves nucleophilic trapping [8]. In the case of **50** and **51**, an alternative to quadrangularin A is 8–10′ resveratrol dimer (**8** and **9**, respectively), which can undergo nucleophilic trapping onto **7** in a fashion similar to cajyphenol A, resulting in regioselective bond formation (Fig. 5). This oligomerization mode, also known as the intramolecular Friedel–Crafts reaction, is insignificant in the biogenesis of resveratrol tetramers.

#### Highly condensed RO

Vaticanols D (**52**), H (**53**), and I (**54**), are among the first identified hexameric resveratrol natural products, which were isolated from two DPs (*Vatica rassak* [16, 18] and *Cotylelobium lanceolatum* [44]) by us in the 2000s (Fig. 6). During parallel studies, various highly condensed ROs (HCRs) ranging from pentamers to octamers, have been discovered in DPs: *Upuna borneensis* [34, 40, 41, 78], *Vateria indica* [32, 78], and four *Vatica* species (*V. pauciflora*, *V. arbiramis*, *V. chinensis*, and *V. bantamensis*) [20, 23, 25]. The intramolecular Friedel–Crafts reaction occurring between two molecules is the mode of common HCRs, where the electron-rich resorcinol of the ROs also react with the QMs generated during the oligomerization of resveratrol. For instance, **52** and **54** are regioisomeric hexamers of the cross coupling of **31** and **23**, which can be regarded as dimeric resveratrol trimers. When the counterpart of the trimeric unit is substituted with **32**, **53** is generated. The other series of HCRs, specifically vaticanols M (**55**, resveratrol hexamer) [25], pauciflorol D (**56**, resveratrol heptamer) [20], vaticanol J (**57**, resveratrol heptamer) [16], and upunaphenol Q (**58**, resveratrol octamer) [78], have a common counter part of **42**, wherein the electron-rich position (C_12b_) undergoes nucleophilic reaction with the respective QMs; that is, **7**, **20**, **23**, and **35**. Compound **58** is regarded as a dimeric molecule of **42**, which is a major element of resveratrol tetramers in DP. Hitherto, the highest oligomerization degree of resveratrol has been achieved with resveratrol octamers, specifically dimeric resveratrol tetramers. Another resveratrol octamer, vateriaphenol A (**59**), can be biosynthesized when the vaticanol B-counterpart in upunaphenol Q is substituted with **39** [31, 32, 78]. This oligomerization mode, which involves the intermolecular trapping of QM intermediates, is common to many resveratrol pentamers that are biosynthesized after resveratrol tetramer (vaticanol B (**42**)) to be reacted with monomeric resveratrol glucoside (piceid (**2**)), resulting in the production of a pentamer, namely upunoside A (**60**) (Fig. 7) [40]. The existence of said aglycons has never been demonstrated.

The similar biosynthetic machinery responsible for the production of a resveratrol pentamer glucoside is hopeaside A (**61**) (Fig. 7) [56]. 2-*C*-β-glucopyranosyl resveratrol (**4**) proceeds by epoxidation; it is prone to attack by nucleophiles, such as the electron-rich arenes of **37**, which produces **61**.

As described above, major HCRs can be produced by the cross coupling of two ROs through an intermolecular Friedel–Crafts reaction in DPs. Unfortunately, little is known about examples of minor HCRs condensed only by radical couplings. The rare example of this is upunaphenol A (**62**) [41], which can be produced via radical coupling between **43** and **5** in the 3–8′ mode and/or **5** and **48** in the 8–10′ mode (Fig. 8).

### RO glucosides

The chemical diversity of ROs in DPs also stems from modification by glucosylation, in which beta-glucopyranosyl groups are introduced prior to the oligomerization of resveratrol units. Collectively, they are referred to as the *O*- and *C*-glucosides of ROs.

#### *O*-Glucosides

In the case of *O*-glucosides of RO (*O–*G–RO), it is evident that **2** is a vital BB in ROs, where, in the majority of cases, one glucopyranose can be found in said molecules. Currently, *O–*G–RO bearing aglycon together with **2** have been isolated from many DPs belonging to different families, such as *Vatica*, *Vateria*, and *Upuna*. The majority of *O–*G–RO co-exist with respective aglycon, such as dimers and hexamers; for instance, in vaticasides A–G [16, 17, 25] and pauciflorosides A–C [21], *O–*G–RO are isolated together with their aglycons. Usually, in DPs, *O-*glucosides are found in less amounts compared with their corresponding aglycons. This suggests that the production of said molecules is the result of **2**, which is introduced to the aforementioned biosynthetic pathway instead of **1**.

**Fig. 9:**
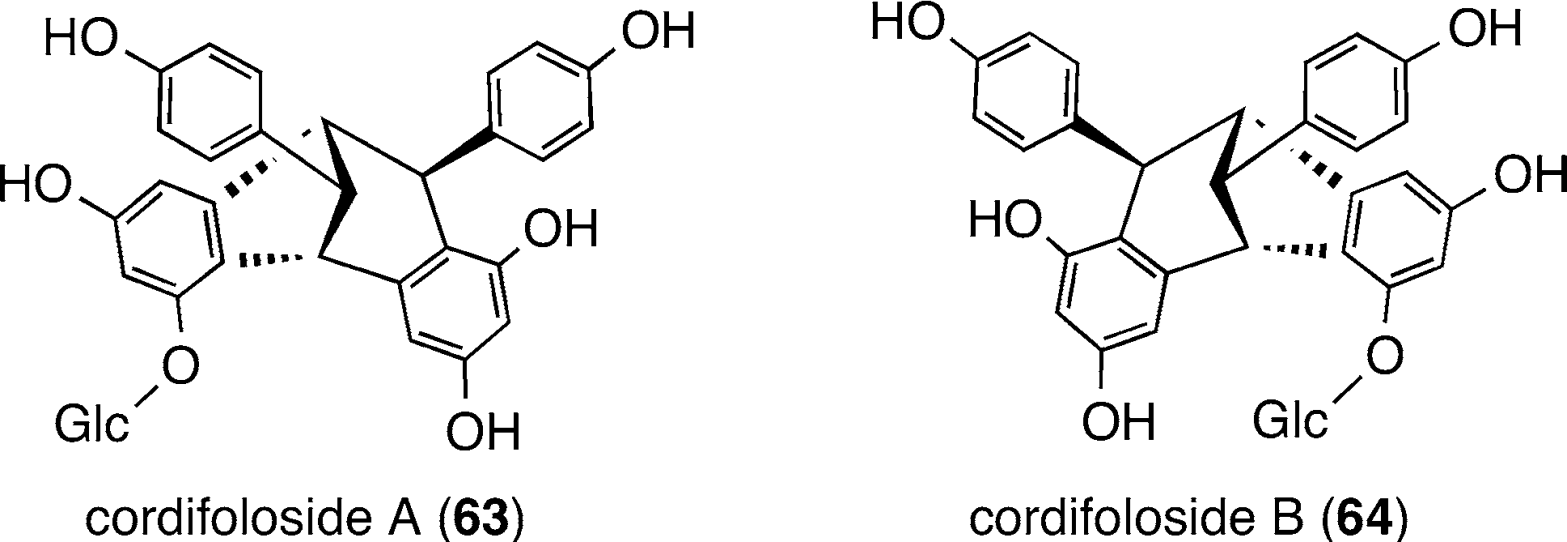
Structures of *O-*glucosides **63** and **64**

So far, *O–*G–RO have been determined to be enantiomerically identical with those of co-existing aglycons; however, the cordifolosides A (**63**) and B (**64**) with enantiomeric aglycons, which are isolated from *Shorea cordifolia* [55], are derived from (−)- and (+)-**9**, respectively (Fig. 9). Moreover, (−)-**9** only co-exists as an aglycon in the plant material. Indeed, this suggests that particular DPs are capable of biosynthesizing *O–*G–RO with enantiomeric aglycons, wherein the aliphatic 8-position of resveratrol is coupled with another aromatic 10′-position of **2**.

#### *C*-Glucosides

With respect to *C*-glucosides of RO (*C-*G–RO), chemical scaffolds have been identified after phytochemical studies on *Shorea* and *Hopea* species. In particular, it has also been demonstrated that the BBs, 4-*C*-β-glucopyranosyl resveratrol (**3**) and **4**, co-exist in the respective species of *Shorea* (*S. hemsleyana* and *S. uliginosa*) and *Hopea* (*H. parviflora* and *H. utilis*). Notably, *C-*G–RO can be produced via their biosynthetic pathways. These are different from those of nonglucosylated ROs, which, according to previous phytochemical studies, have fewer structural correlations between *C-*G–RO and RO.

**Fig. 10:**
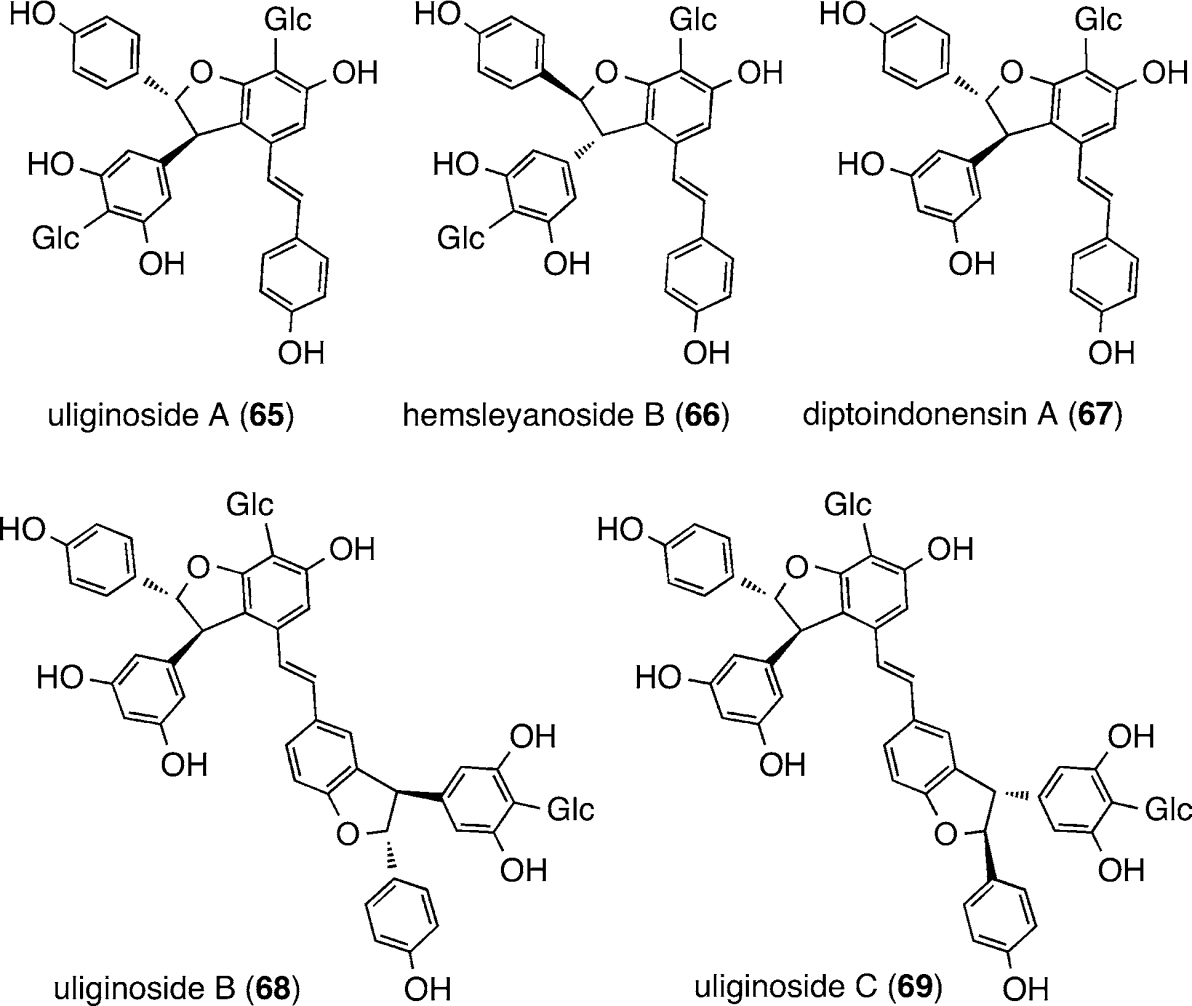
Structures of *C-*glucosides with BB of 4-*C*-β-glucopyranosyl resveratrol

For example, phytochemical investigations of the extract of the stem bark of *S. hemsleyana* failed to isolate the nonglucosylated derivatives of hemsleyanosides A–F (*C-*G-RO) and the *C*-glucosylated derivatives of hemsleyanols A–E (RO) [48–50]. Another crucial aspect of their structural diversity is that C–C bond formation in the regioselective 8–10′ mode proceeds in an non-enantioselective fashion when the aliphatic 8-position of **3** is coupled to another aromatic 10′-position; this can be seen in the isolation of uliginoside A (**65**: C_8a_(*R*)) and hemsleyanoside B (**66**: C_8a_(*S*)) from *S. uliginosa* (Fig. 10) [51, 53]. Interestingly, the 8–10′ bond formation between the 8-position of resveratrol and the 10′-position of **3** proceeds enantioselectively; moreover, the *C*-glucoside of (−)-ε-viniferin (diptoindonensin A (**67**) [79]) together with its biosynthetically downstream *C-*G-resveratrol trimers (uliginosides B (**68**) and C (**69**)) have been identified [51, 53]. These trimers (**68** and **69**) also have antipodal dihydrobenzofuran moiety, which is produced after non-enantioselective 3–8′ bond formation, as seen in **65** and **66**.

**Fig. 11:**
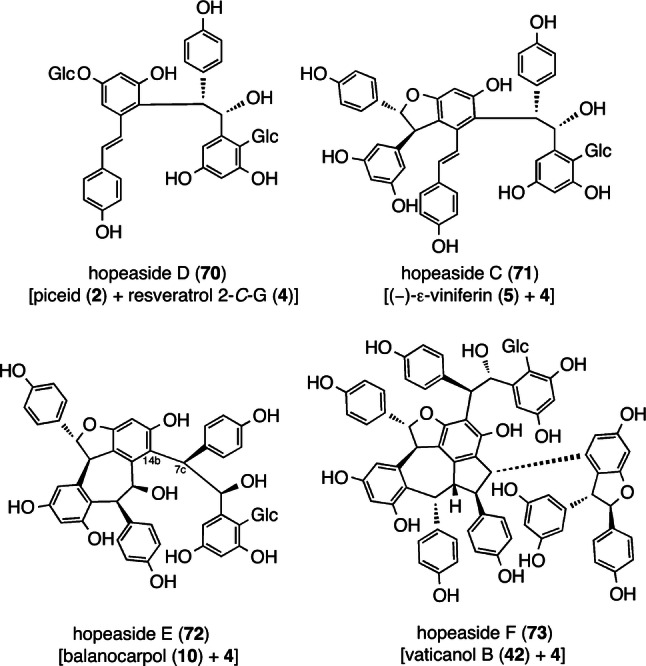
Structures of *C-*glucosides with BB of 2-*C*-β-glucopyranosyl resveratrol

The chemical diversity of *C-*G–RO with BBs of **3** is the result of the regioselective intramolecular radical coupling and cyclization pathways. Typically, intramolecular cyclization or tautomerization of an intermediate para-quinone methide, which is also described for resveratrol dimers (Fig. 2), outcompetes most intermolecular processes. Some of the resultant dimers undergo further radical coupling through modes of 3–8′, 8–8′, and 8–10′(14′) to produce high-order oligomers with **3**. Alternatively, it is assumed that the BBs of **4** are not displaced from **3** in the metabolic pathway; this is supported by the fact that no fused-ring systems involving **4** have been found. Instead, it is acceptable to assume that intermolecular functionalization by oxide of **4** occurs, wherein the electron-rich position of ROs reacts with their 7-position. Indeed, it is assumed that dimer (hopeaside D (**70**)), trimers (hopeasides C (**71**) and E (**72**)), and pentamers (hopeasides A (**61**), B (C_7e_–epimer of **61**), and F (**73**)) are produced after the crossed coupling of **4** with monomer (**2**), dimers (**5** and **10**), and tetramers (**37** and **42**), respectively (Fig. 11) [56, 59, 60].

### ROs as sources of chemical diversification

Increased structural diversity is further produced by minor ROs and subsequent modifications, such as additional oxidation events and/or the attachment of additional atoms or groups. In addition, diversified ROs are further produced by dearomatization, tautomerization, substituent rearrangement, and so forth. Although the absolute quantity of said modified ROs is much less than those of precursor molecules, the isolation of such derivatives indicates a vast array of chemical RO scaffolding. Accordingly, we focus on such molecules to expand on the aspects of ROs.

#### Dearomatized, rearranged, and/or oxidatively cleaved ROs

The isolation of the dearomatized, rearranged, and/or oxidatively cleaved ROs demonstrates another level of chemical diversity. The first discovery of a dearomatized RO was achieved by the isolation of gnetin A (resveratrol dimer) with dearomatized resorcinol from *Gnetum leyboldii* (Gnetaceae) [80]. A subsequent report of leachianol C (resveratrol trimer) in *Sophora leachiana* (Fabaceae) by Ohyama et al. [81] was the first to identify dearomatized phenol in ROs. The finding of gnetin A and leachianols A and B [82], along with the isolation of kobophenol B (resveratrol tetramer) from *Carex pumila* (Cyperaceae) reported by Kawabata et al. [83], advanced the notion that the resveratrol units in ROs are condensed not only by regioselective radical–radical couplings followed by regiodivergent Friedel–Crafts cyclization, but also by a formal dearomative [3 + 2] annulation, forming the bicyclo[3.2.1]octedione. Alternatively, the dearomatized phenol structure in leachianol C can be regarded as the result of dearomative [3 + 3] annulation in forming the bicyclo[3.3.1]nonedione. The aforementioned resveratrol tetramer with the dearomatized phenol, **38** [33, 54], is formed by a different mechanism, where asymmetric dearomatization undergoes intramolecular-stereoselective cyclization (but not intermolecular-annulation reaction) in the biogenetic course.

**Fig. 12:**
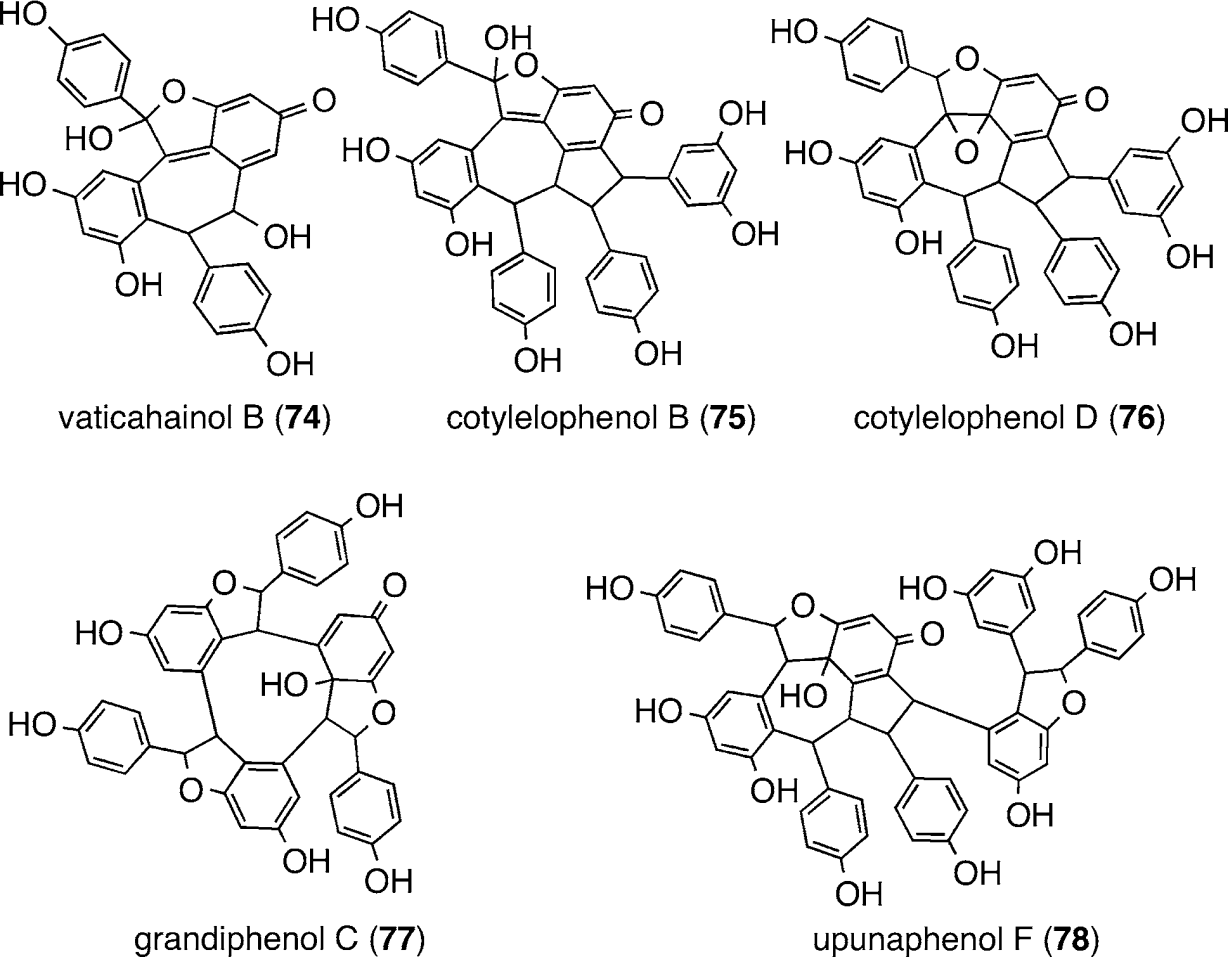
ROs with a dearomatized resorcinol ring

The other examples of dearomatized ROs are vaticahainol B (**74**) (resveratrol dimer) [84], cotylelophenols B (**75**) and D (**76**) (resveratrol trimers) [43, 44], grandiphenol C (**77**) (resveratrol trimer) [45], and upunaphenol F (**78**) (resveratrol tetramer) [39] with dearomatized resorcinol, which, after oxidation, can be formed from the respective precursor molecules, **12**, **31**, **28**, and **42** (Fig. 12).

**Fig. 13:**
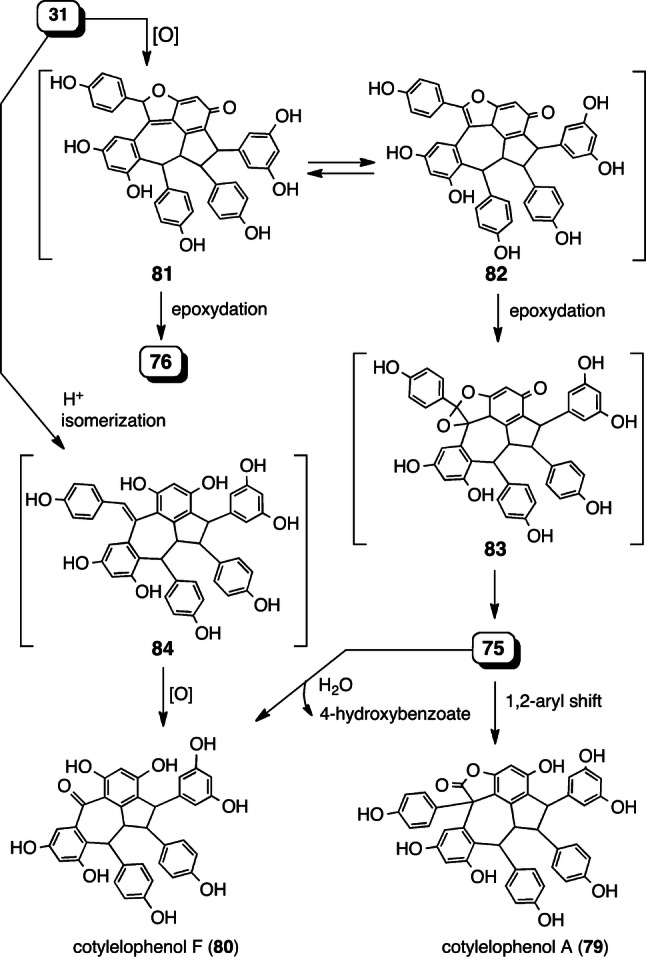
Proposed biogenic relationship between **31** and the dearomatized, rearranged, and/or oxidatively cleaved metabolically downstream products

The further isolation of rearranged 10–8′ trimers bearing a dibenzocycloheptane ring as well as cotylelophenols A (**79**) and F (**80**) [43, 44] provides insight into the associated biogenic relationships and plausible intermediates (**81**–**84**) (Fig. 13). Indeed, the oxidation of **31** may produce the hypothetical intermediate **81**, since **76** is a product of its epoxydation. Following the isomerization of **81**, benzofuran-6(3a*H*)-one **82** can also undergo epoxidation to generate intermediate **83**, which is prone to isomerize to **75**. A 1,2 aryl migration from **75** yields **79**. Upon water trapping, **75** can undergo oxidative cleavage of its C_7a_–C_8a_ bond, followed by the hydrolysis of 4-hydroxybenzoate to obtain **80**. Alternatively, **80** can possibly be derived from oxidative cleavage of another olefin of a plausible intermediate, specifically **84**, which can be derived from the isomerization of **31**.

**Fig. 14:**
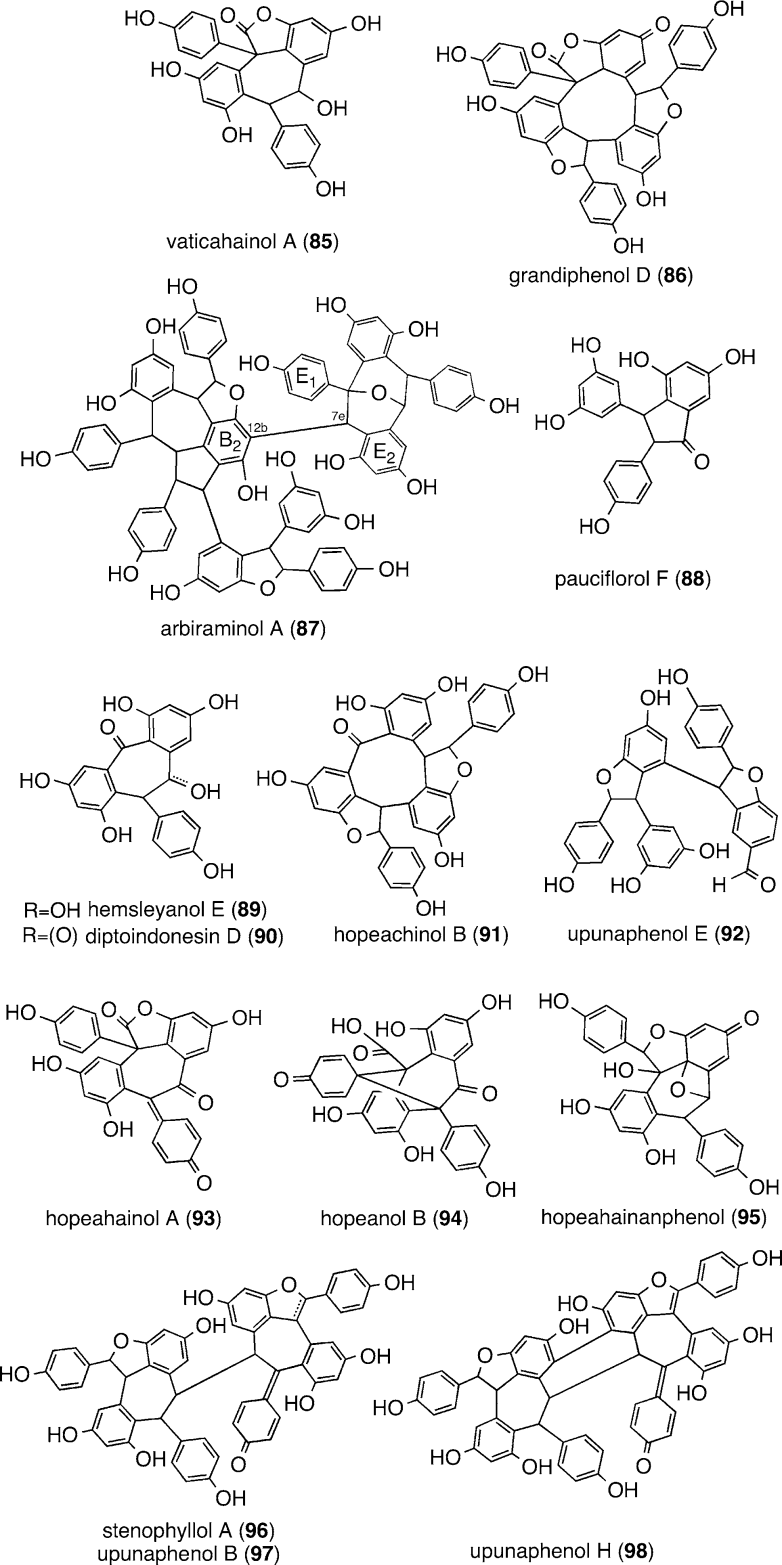
Structures of ROs produced via oxidation, rearrangement, and dearomatization

Several rearranged and/or oxidatively cleaved resveratrol trimers have been isolated in DPs. A similar set of 1,2 aryl migration has been observed for the 8–10′ dimer and 8–10′ trimer, wherein the 1,2 aryl rearrangement of **74** and **28**results in vaticahainol A (**85**) [84] and grandiphenol D (**86**) [45], respectively (Fig. 14). The rearranged aryl group is also exemplified by the minor resveratrol hexamer, arbiraminol A (**87**) [23], which is presumably obtained owing to the 1,2 aryl rearrangement of ring E_1_. Pauciflorol F (**88**) [20], hemsleyanol E (**89**) [48], hopeachinol B (**91**) [85], and **92** form due to the oxidative cleavage of **8**, **74**, caraphenol A [86], and **27** (or **47**), respectively. Diptoindonesin D (**90**) [87] and **89** differ only in the oxidation state at the C_8b_ benzylic position. Further oxidation of **85** affords the keto-conjugated QM, hopeahainol A (**93**) [88], which is prone to lactone-ring hydration and, to obtain hopeanol B (**94**), goes through intramolecular 5-exo-trig Friedel–Crafts cyclization [86]. The oxidation of **10**, which is followed by successive oxidative dearomatization, may result in the formation of hopeahainanphenol (**95**) [89].

An array of highly oxidized and structurally rearranged derivatives of **39** is also unique to DPs, which can be derivatized from the oxidative product of **39**, which is stenophyllol A (**96**) (Fig. 14) [71]. When **96** further undergoes two- and four-electron oxidation, the benzofuran derivatives, upunaphenols B (**97**) [38] and H (**98**) [37], are respectively obtained, resulting in the extension of the conjugated system. Electron-mediated intramolecular C–C bond formation for **98** can also be seen in the biogenesis of **13** (Fig. 2).

#### Hetero-coupled ROs

**Fig. 15:**
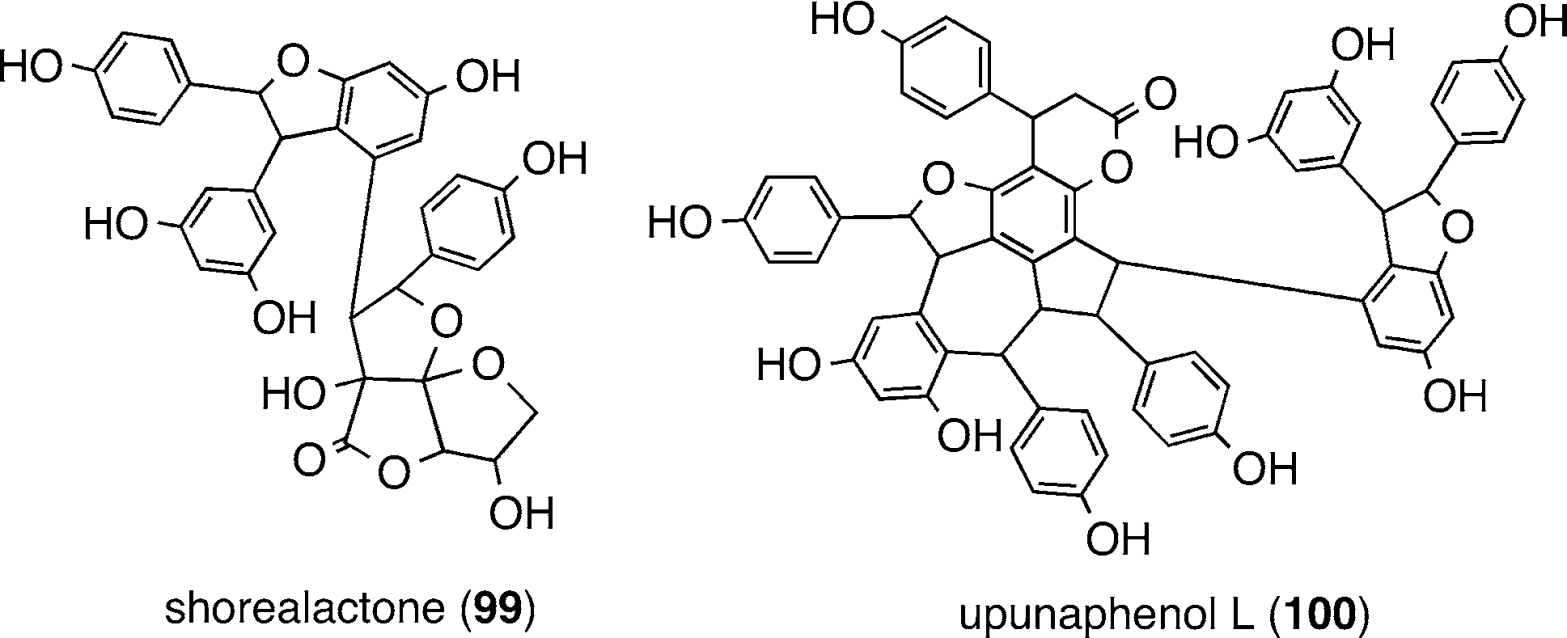
Structures of hetero-coupled ROs

In 2000, our team discovered and reported the structure of shorealactone (**99**) (Fig. 15), the ascorbyl-resveratrol dimer derivative isolated from *Shorea hemsleyana*, which is the first example of hetero-coupled ROs [47, 90]. The connectivity of the tricyclic-tetrahydrofuran core was the first instance found in naturally occurring polyphenols. Upon oxidation of **5** and ascorbic acid, the hypothetical QM intermediate, **5B**, and monodehydroascorbic acid form a C–C bond, which is followed by concerted regioselective cyclization pathways to generate **99**. Later, an identical compound was isolated from the heartwood of *Shorea laeviforia* by Hirano et al. and given a second name; namely, laevifonol [91].

Upunaphenol L (**100**) is the first instance of lignostilbenoids in DPs, which is formed by the fusion of the electron-rich arene of **42** and a phenylpropan unit [36]. It is assumed that ROs can undergo hetero coupling when the other reactive radical species exist.

### RO stereochemistry

It is an interesting challenge to analyze the stereostructure of ROs. The central obstacles to elucidating the relative configuration consist of the following: poor prognosticators of the vicinal coupling constants required for configurational elucidation of the bicyclo five- and seven-membered ring systems; difficulties in affirming NOEs and ROEs in conventional two-dimensional nuclear magnetic resonance (NMR) spectra (NOESY and ROESY, respectively) due to the duplicated proton signals in determining substituent orientation and in elucidating the configuration and conformation of C_2_ molecules; the existence of the chiral axis. In addition, diminished NMR-signal intensity due to coalescence is also problematic when variable temperature NMR (VT-NMR) is not available. Another crucial property is anisotropy frequently observed in proton NMR (^1^H-NMR), the analysis of which in combination with two-dimensional NMR data and three-dimensional molecular modeling would help in elucidating the relative configuration, especially when partial structures are connected through the chiral axis.

As described above, DPs produce a number of OS analogues, which typically possess a common skeleton of 1,2-diaryl-dihydrobenzofuran stemming from (−)-**5**. This RO stereochemical homogeneity suggests that the downstream biosynthetic product of (−)-**5** has the same absolute configuration in the 1,2-diaryl-dihydrobenzofuran skeleton. Alternatively, other types of ROs exist in this plant family, such as **63**–**66**) (Figs. 9, 10). The fact that they bear antipodal stereochemistry in each 1,2-diaryl-dihydrobenzofuran and non-heterocyclic bicyclo[3.2.1] system demonstrates the need for various approaches in combination with the solid physicochemical approach to determine the absolute configuration, instead of speculating plausible biosynthetic precursors, such as (−)-**5**. Although the number of reports on RO stereochemistry is increasing, their absolute configuration is yet to be determined.

#### Distereoisomerism

**Fig. 16:**
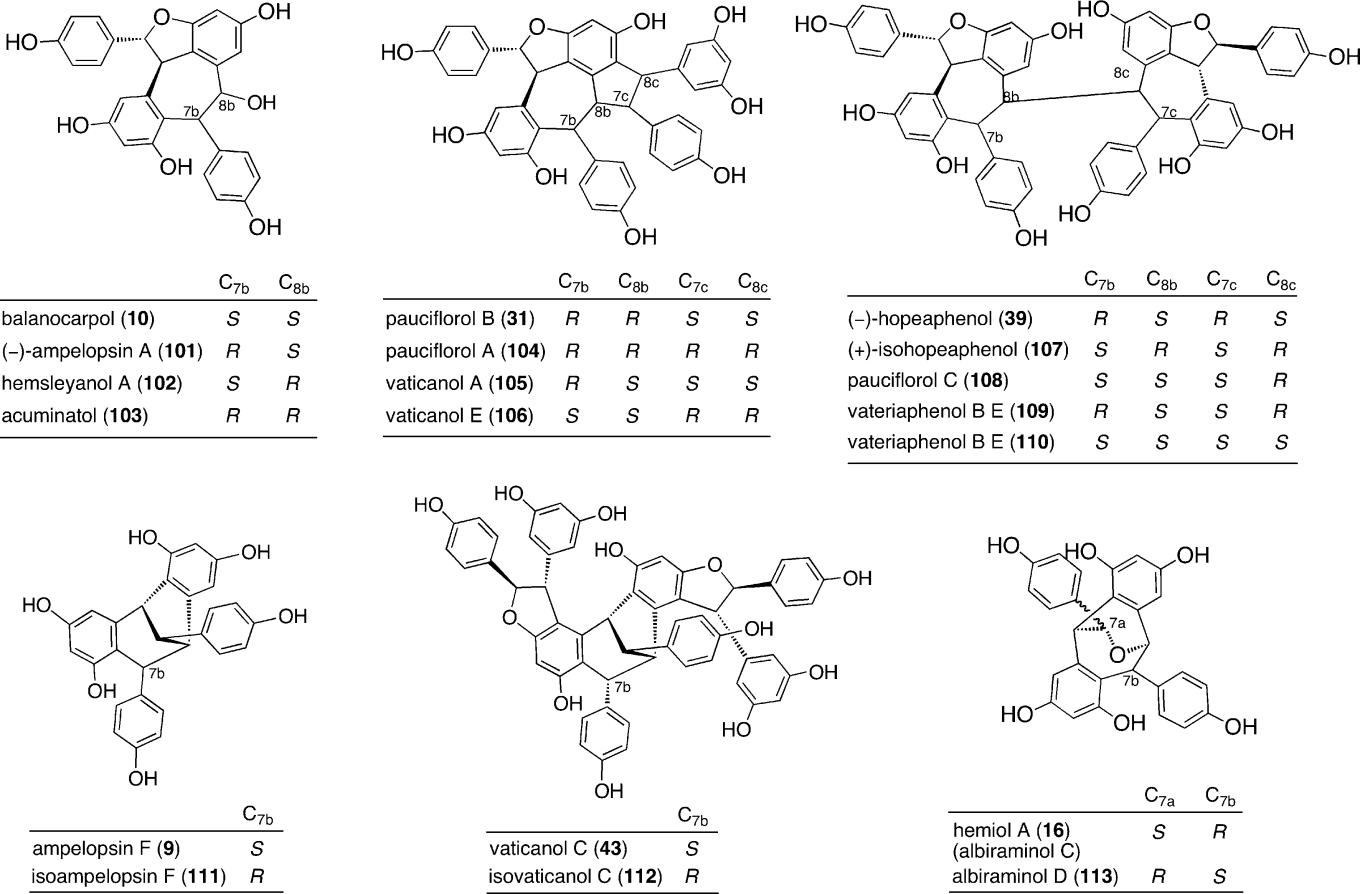
Diastereomeric ROs

It is known that distereoisomerism is a central factor in the structural diversity of ROs in DPs. In essence, ROs with various configurations can be attributed to the asymmetric carbons stemming from the C_7_ and C_8_ position of resveratrol. Isolated compounds exhibit diastereoisomerism as well as several configuration patterns, such as the four diastereomeric oxidized 8–10′ dimers, **10** [66], (−)-ampelopsin A (**101**) [58], hemsleyanol A (**102**) [50], and acuminatol (**103**) [92] (Fig. 16). These present the possible configurations of the two asymmetric carbons, C_7b_ and C_8b_, and can be differentiated according to the chemical shifts for aliphatic protons. The co-existence of **10** and **101** in several DPs reinforces the idea that the initial epoxidation step takes place in an enantioselective fashion, followed by the non-stereoselective 7-exo-trig Friedel–Crafts cyclization. The example of diastereomeric trimers are best shown by the 10–8′ trimers, pauciflorols A (**104**) and B (**31**) [21] and the vaticanols A (**105**) [19] and E (**106**) [17]. Indeed, the generation of their biogenetic intermediate, **1B**–**5E** QM (**13**), proceeds through the non-enantioselective 10–8′ coupling of (−)-**5** and resveratrol, which enables the co-existence of both the configurations of the C_8c_ asymmetric carbon (C_8c_(*R*): **104** and **106**; C_8c_(*S*): **31** and **105**). The epimeric **1B**–**5E** QM intermediate (**13**) follows successive non-stereoselective Friedel–Crafts cyclization (5-exo-trig and 7-exo-trig cycles), thereby obtaining said diastereoisomeric divergent derivatives. In the case of tetrameric ROs, it is understandable that diastereoisomers stem from the non-enantioselective 8–8′ coupling of two molecules of (−)-**5**, resulting in the production of a series of diastereoisomeric C_2_ molecules, specifically (−)-**39** [7], (+)-isohopeaphenol (**107**) [30], pauciflorol C (**108**) [21], and vatreriaphenols B (**109**) [31] and C (**110**) [30]. The relative configuration of these diastereomers can be determined using the data obtained from the differential NOE, NOESY, and/or ROESY experiments.

#### Rotational isomerism

**Fig. 17:**
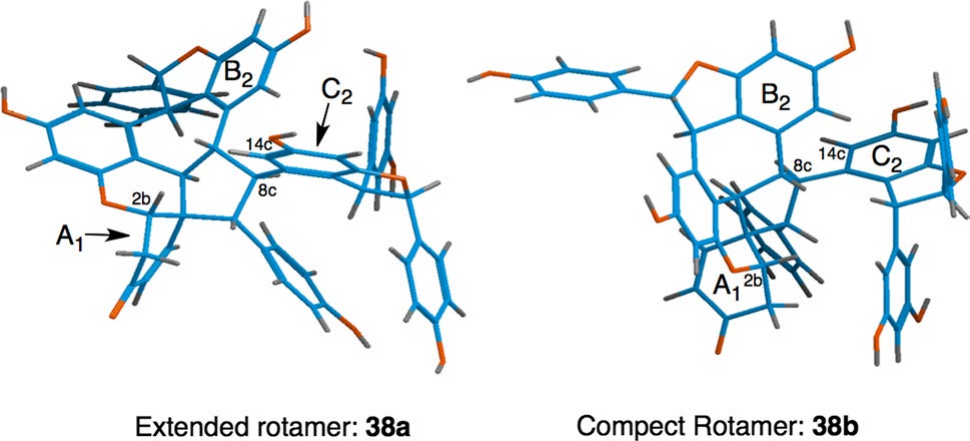
Energy-minimized conformations of the two atropisomer structures of hetero-coupled ROs

Compound **38** is a precedent of the atropisomeric ROs having two configurationally stable two rotational isomers (extended rotamer **38a** and compact rotamer **38b**) (Fig. 17) at an ambient temperature in the NMR time scale [33, 54]. The separation of each conformer is unaccomplished due to exchangeable properties through the chiral axis. Although its peracetate only has one conformer, the deacetylated product has signal duplications due to atropisomerism. Changes in the ratio of the two conformers and the various solvents of the VT-NMR as well as the cross peaks due to conformational exchange observed in the NOESY experiments can be attributed to rotational isomerism. The complete and unequivocal assignment of proton and carbon resonances of the two rotational isomers is demonstrated through structural analysis. The rotational state of the rotamers can be defined using NOESY experiments, which show correlations between H_8c_ and H_14c_. Accordingly, it is possible to differentiate the two rotamers. Each conformation is supported by the anisotropy that is explained by the different chemical shifts of H_2b_ and H_14c_ in the two rotamers. Decisive evidence for the absolute configuration can be obtained by the acid-catalyzed rearrangement of **38**, resulting in the formation of a monoalkyl ether of the known resveratrol tetramer; namely, (+)-**107**.

The second instance of an atropisomeric RO is **72** [59]. Because **72** has covalent C–C bonds connecting two partial structures (i.e., **10** and **4**)), the configurational relationship between them and the conformational determination due to the rotational isomerism are critical issues in the stereostructure analysis. The NMR spectra in MeOH-*d*_4_ show multiplicity, which possibly stems from the rotational isomerism through the chiral axis, C_14b_–C_7c_, displaying signals due to a major conformer and a minor one. Moreover, when the material recovered from the MeOH-*d*_4_ solution is re-dissolved in acetone-*d*_6_, the ^1^H NMR spectrum only shows major conformers. These results indicate that **1** undergoes conformational isomerism in MeOH, an observation that is further confirmed by the NOESY correlations by chemical exchange for the aromatic signals. Another important issue associated with the determining the absolute configuration of **72** is the comparative-configurational analysis, which is conducted using the β-d-glucopyranosyl group and has been previously demonstrated with respect to **71** and **70** [56].

#### Steric hindrance upon rotation of aromatic rings

**Fig. 18:**
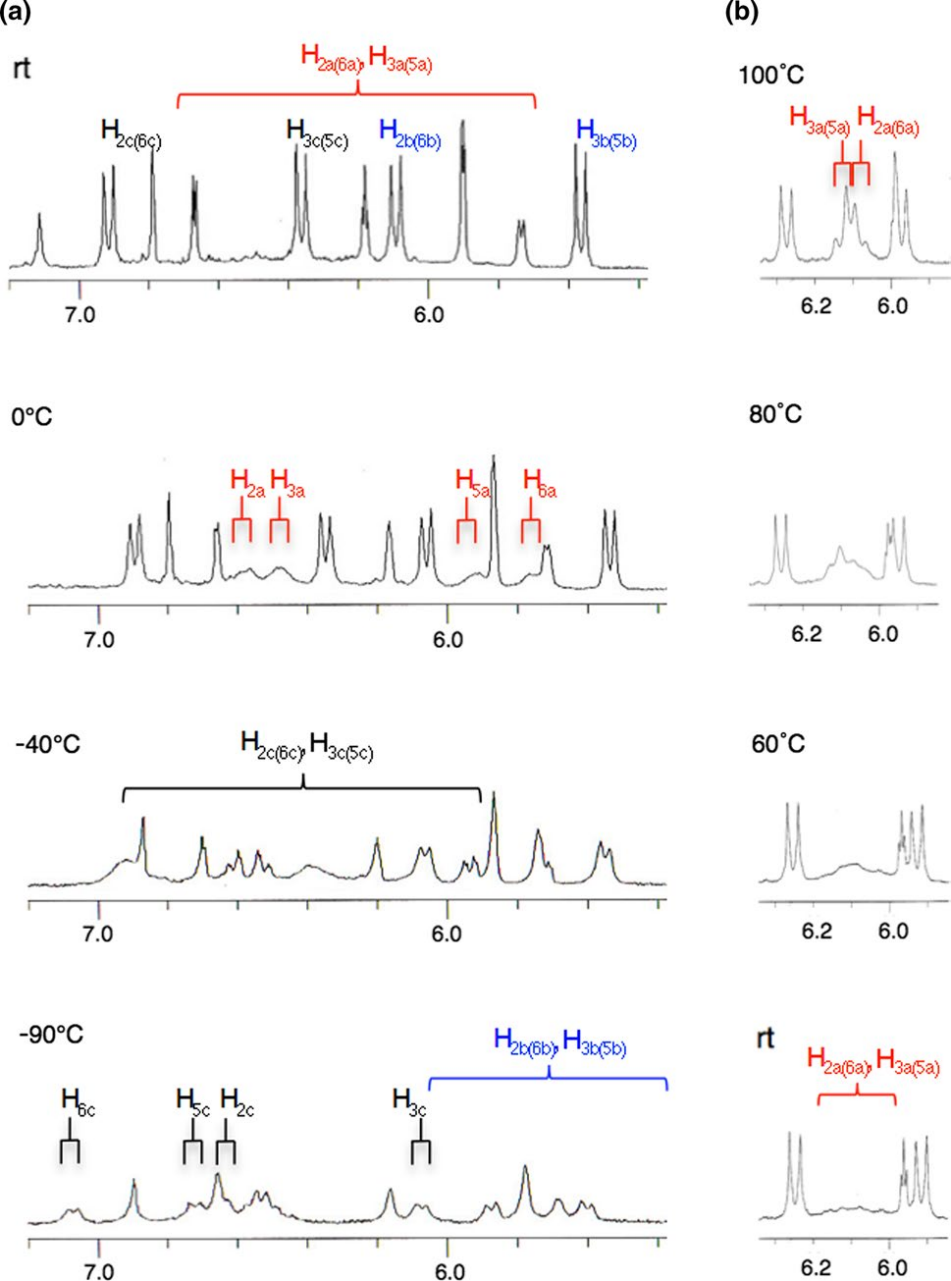
^1^H-NMR spectra (300 MHz) of **79** at variable temperatures (*a)* in acetone-*d*_6_; rt—90 °C and (*b)* in DMSO-*d*_6_; rt—100 °C

**Fig. 19:**
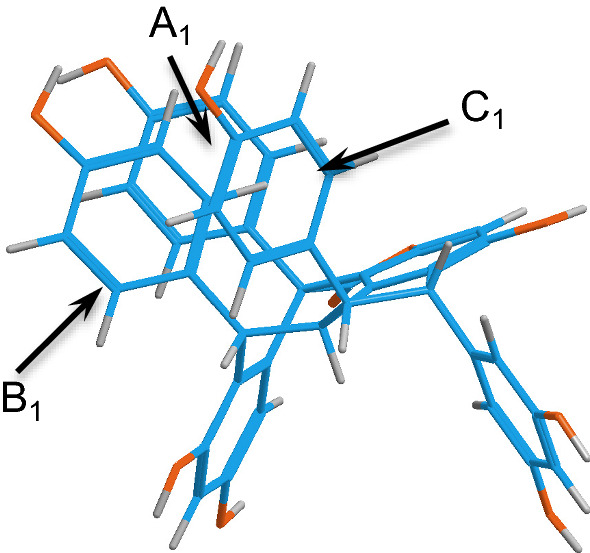
Stereostructure of **79**

The restricted rotation of aromatic substituents is a well-recognized property, which provides crucial information in elucidating configuration and conformation. Compound **32**, for example, the two set of aromatic protons (H_2c_/H_6c_ and H_3c_/H_5c_) in a 4-hydroxyphenyl group, is non-equivalent due to the hindered rotation about the C–C bond (C_1c_–C_7c_), which can be seen in the NMR with four independent ^1^H and ^13^C broad signals (H_2c_, H_3c_, H_5c_, and H_6c_; C_2c_, C_3c_, C_5c_, and C_6c_) [16]. The other examples are presented by isoampelopsin F (**111**: C_7b_-epimer of **9**) [93], isovaticanol C (**112**: C_7b_-epimer of **43**) [21], **79** [44], and arbiraminol D (**113**: C_7a,7b_-diastereomer of **16**) [22]. The NMR behavior of **79** is particularly significant because all 4-hydroxyphenyl groups are rotationally restricted, wherein complete structural elucidation is achieved by the aid of the VT-NMR experiment, as was done for **32** (Fig. 18). The energy-minimized structure suggests that the higher field shifts of aromatic protons on rings A_1_–C_1_ can be explained by the anisotropic effects caused by the neighboring rings (Fig. 19). For example, at –0 °C, where H_5a_ and H_6a_ can be observed at δ 5.90 and 5.70, the higher field shifts are caused by the effect of ring B_1_. The effects of both rings (A_1_ and C_1_) results in the higher field shifts of H_2b,6b_ and H_3b,5b_. At –90 °C, where the aromatic proton on ring C_1_ is observed as four separated signals, the higher field shift of H_3c_ can be observed at δ 6.08, which can be attributed to the anisotropic effect of ring B_1_. As can be seen in the structural elucidation of **79**, the exact understanding of the coasealence caused by the hindered rotation of aromatic rings, as well as the accompanying anisotropic effects on aromatic protons, helps in determining the relative configuration and conformation of ROs.

When the partial structures connected through the C–C bond increase in size, the rotational barriers also increase, which results in a stable conformer. Examples of this can be seen in **42** and **37** with the 3-(3,5-dihydroxyphenyl)-6-hydroxy-2-(4-hydroxyphenyl)-2,3-dihydrobenzofuran-4-yl group (1,2-diaryl-dihydrobenzofuran) connected to the dibenzobicyclo[5.3.1]octadiene core, where H_8c_ and H_14c_ are situated in syn orientation. The hindered rotation and conformational stability in such molecules can also be enhanced by attractive forces, such as CH–π and OH–π interactions [94, 95].

**Fig. 20:**
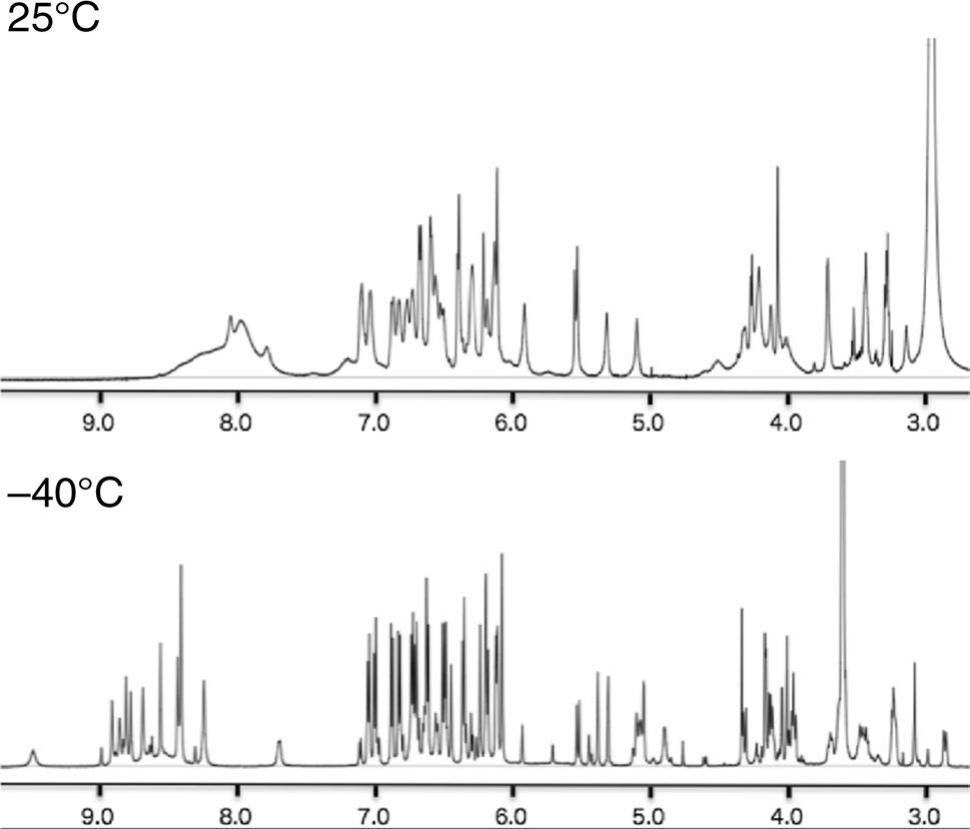
^1^H-NMR spectra (600 MHz) of **114** in acetone-*d*_6_ at 25 °C and − 40 °C

**Fig. 21:**
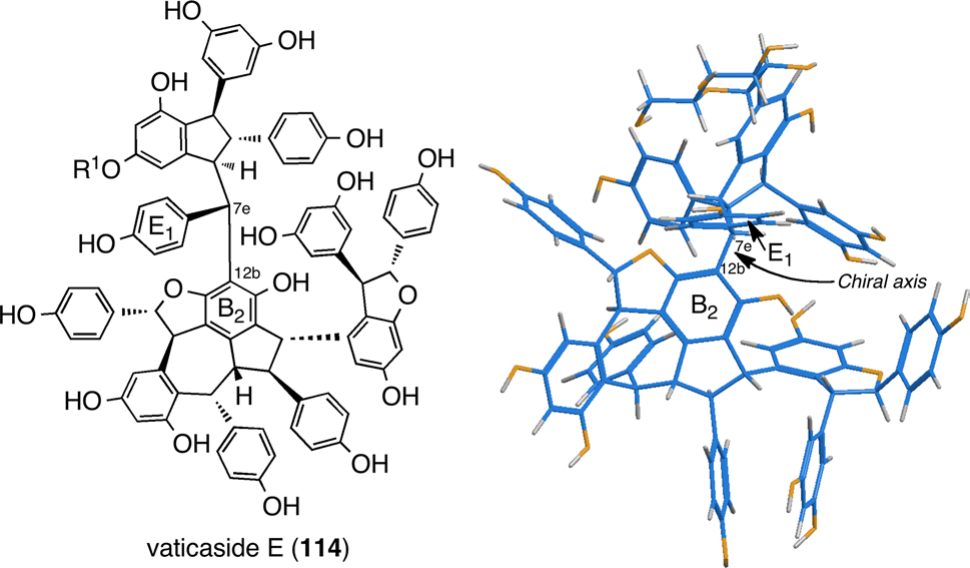
Stereostructure of **114**

Alternatively, the particular alignment of partial structures could weaken the aforementioned rotational restrictions, which, in turn, can causing coasealence as well as difficulties in structural elucidation. Compound **55** exhibited broad signals in the entire region due to unstable conformation at ambient temperatures [25]. Indeed, in the spectrum, reducing the temperature results in a change in the signal features to clear; some substituents did not display signals. The significant features consist of signals for aromatic protons for ring E_1_ in various conditions (temperatures and solvents) as well as the completely overlapping methine signals. Successful isolation of glucosides of **55** (vaticasides E (**114**) and F [25]) finally enable spectroscopic-data analysis, where clear NMR signals can be attributed to the weakened coasealence due to the enhanced hindered rotation of the C_12_–C_7e_ bond, which, in turn, results in the successful determination of **55** (Figs. 20, 21).

#### Approaches to determine absolute configuration

Some ROs with absolute configurations determined by different approaches have been summarized in the existing literature [78]: X-ray crystallographic analysis of their chemical derivatives using anomalous scattering of the bromine atom(s) ((−)-**39** [7] and **99** [47]; the comparison of optical rotation and/or circular dichroism ((+)- and (−)-**5** [96], (+)-**39** [97], **65** and **66** [52, 53], **63**) and **64**) [55], and modified Mosher′s method (**85**) [84]; the comparison of the experimental and theoretical ECD spectra (**42**) [84]; the application of the olefin-cleavage strategy to a known compound to obtain ECDs of the newly separated products (**68** and **69** [52, 53] as well as laetevirenol D [98]); the regioselective and stereospecific transformations of a hypothetical biogenetic precursor, (+)-**5** ((+)-**101** [99], (+)-vitisin A [100]); the acid-catalyzed skeletal conversion to obtain monoalkyl ether of the known derivative (**38**) [33]; assignment based on the comparison of the absolute configuration of the d-β-glucopyranosyl group (**70**–**72**)) [56, 59, 60]; comparison of experimental and theoretical electronic circular-dichroic spectra of the dehydroxylated derivative (( −)-**31**) [101]; comparative study using ECD with the help of the ECD of known compounds with previously determined absolute configurations (**58** and **59** [78]). Currently, however, the absolute configurations of many ROs are yet to be determined. This is because, in typically cases, ROs are neither crystalline nor secondary alcohols, which is to say they are unsuitable for general methodologies. The application of a comparative study using an ECD database and X-ray analysis using porous complexes [102] is promising with respect to determining the absolute configuration of RO scaffold; however, this depends on a reliable chemical library. To be sure, the object of this review was not to provide a comprehensive example of the absolute configuration determination of ROs. Accordingly, a forthcoming review will be directed toward a better understanding of various methods to solve the issue in question.

## Concluding remarks

Even though a considerable amount of knowledge is available with respect to the structural diversity of ROs in DPs (particularly, the 8–8ʹ and 8–10ʹ linked compounds), compounds with other link modes have not yet been comprehensively studied. This includes defining versatile structural motifs stemming from minor couplings and structural modifications (i.e., in terms of introduction of *O*-atom(s), dearomatization, rearrangement, tautomerization, and a hetero-coupling with other BBs), as well as deducing further stereochemical diversity in the chemical pool. Much work remains in clarifying the differences in physicochemical properties among the diverse stereoisomers that arise from enantiomerism, diastereomerism, and atropisomerism; these will be the subject of future work, as will be defining further RO structural diversity.
