# Correction to: Resveratrol oligomer structure in Dipterocarpaceaeous plants

**DOI:** 10.1007/s11418-020-01416-7

**Published:** 2020-05-30

**Authors:** Tetsuro Ito

**Affiliations:** 1 Laboratory of Pharmacognosy, Gifu Pharmaceutical University, 1-25-4 Daigaku-nishi, Gifu, 501-1196 Japan; 2 Present Address: Laboratory of Pharmacognosy, Department of Pharmacy, Faculty of Pharmacy, Gifu University of Medical Science, 4-3-3 Nijigaoka, Kani, Gifu, 509-0293 Japan

## Correction to: Journal of Natural Medicines 10.1007/s11418-020-01412-x

The article Resveratrol oligomer structure in Dipterocarpaceaeous plants, written by Tetsuro Ito, was originally published electronically on the publisher’s internet portal on 30 April 2020 without open access. With the author(s)’ decision to opt for Open Choice the copyright of the article changed on 22 May 2020 to © The Author(s) 2020 and the article is forthwith distributed under a Creative Commons Attribution 4.0 International License (https://creativecommons.org/licenses/by/4.0/), which permits use, sharing, adaptation, distribution and reproduction in any medium or format, as long as you give appropriate credit to the original author(s) and the source, provide a link to the Creative Commons licence, and indicate if changes were made.

The original article was updated.

